# Molecular detection of *Coxiella* spp. in ticks (Ixodidae and Argasidae) infesting domestic and wild animals: with notes on the epidemiology of tick-borne *Coxiella burnetii* in Asia

**DOI:** 10.3389/fmicb.2023.1229950

**Published:** 2023-07-27

**Authors:** Abid Ali, Muhammad Kashif Obaid, Mashal M. Almutairi, Abdulaziz Alouffi, Muhammad Numan, Shafi Ullah, Gauhar Rehman, Zia Ul Islam, Sher Bahadar Khan, Tetsuya Tanaka

**Affiliations:** ^1^Department of Zoology, Abdul Wali Khan University Mardan, Mardan, Khyber Pakhtunkhwa, Pakistan; ^2^Department of Pharmacology and Toxicology, College of Pharmacy, King Saud University, Riyadh, Saudi Arabia; ^3^King Abdulaziz City for Science and Technology, Riyadh, Saudi Arabia; ^4^Department of Biotechnology, Abdul Wali Khan University Mardan, Mardan, Khyber Pakhtunkhwa, Pakistan; ^5^College of Animal Husbandry and Veterinary Sciences, Abdul Wali Khan University Mardan, Mardan, Khyber Pakhtunkhwa, Pakistan; ^6^Laboratory of Infectious Diseases, Joint Faculty of Veterinary Medicine, Kagoshima University, Kagoshima, Japan

**Keywords:** ticks, *cox1*, *Coxiella burnetii*, *GroEL*, domestic and wild animals, Pakistan

## Abstract

Tick-borne *Coxiella* spp. are emerging in novel regions infecting different hosts, but information regarding their occurrence is limited. The purpose of this study was the molecular screening of *Coxiella* spp. in various ticks infesting goats, sheep, camels, cattle, wild mice, and domestic fowls (*Gallus gallus domesticus*) in various districts of Khyber Pakhtunkhwa, Pakistan. Morphologically identified tick species were confirmed by obtaining their *cox1* sequences and were molecularly screened for *Coxiella* spp. by sequencing *GroEL* fragments. Almost 345 out of 678 (50.9%) hosts were infested by nine tick species. Regarding the age groups, the hosts having an age >3 years were highly infested (192/345, 55.6%), while gender-wise infestation was higher in female hosts (237/345, 68.7%). In collected ticks, the nymphs were outnumbered (613/1,119, 54.8%), followed by adult females (293/1,119, 26.2%) and males (213/1,119, 19.7%). A total of 227 ticks were processed for molecular identification and detection of *Coxiella* spp. The obtained *cox1* sequences of nine tick species such as *Hyalomma dromedarii, Hyalomma anatolicum, Haemaphysalis cornupunctata, Haemaphysalis bispinosa, Haemaphysalis danieli, Haemaphysalis montgomeryi, Rhipicephalus haemaphysaloides, Rhipicephalus microplus*, and *Argas persicus* showed maximum identities between 99.6% and 100% with the same species and in the phylogenetic tree, clustered to the corresponding species. All the tick species except *Ha. danieli* and *R. microplus* were found positive for *Coxiella* spp. (40/227, 17.6%), including *Coxiella burnetii* (15/40, 6.7%), *Coxiella* endosymbionts (14/40, 6.3%), and different *Coxiella* spp. (11/40, 4.9%). By the BLAST results, the *GroEL* fragments of *Coxiella* spp. showed maximum identity to *C. burnetii, Coxiella* endosymbionts, and *Coxiella* sp., and phylogenetically clustered to the corresponding species. This is the first comprehensive report regarding the genetic characterization of *Coxiella* spp. in Pakistan's ticks infesting domestic and wild hosts. Proper surveillance and management measures should be undertaken to avoid health risks.

## Introduction

Ticks are hematophagous ectoparasites, actively contributing to transmitting infectious agents to wild and domestic animals and humans (De la Fuente et al., 2017). Numerous ticks act as distinguished vectors and reservoirs for various pathogens, including bacteria causing rickettsiosis, anaplasmosis, Lyme disease, viruses such as Powassan, and protozoan agents such as *Theileria* spp. and *Babesia* spp. (De la Fuente et al., 2017; Karim et al., 2017; Rochlin and Toledo, 2020; Ali et al., 2021). Aside from transmitting various infectious agents, ticks are hosts to many endosymbionts and a diversified microbiome (Špitalská et al., 2018).

Among the bacterial genus *Coxiella* having one pathogenic species, *Coxiella burnetii* is a Gram-negative obligate intracellular bacterium distributed worldwide except in New Zealand and French Polynesia (Musso et al., 2014; Eldin et al., 2017). Common reservoirs of *C. burnetii* are domestic mammals, including cattle, sheep, goats, and camels as well as reptiles, birds, and ticks (Anderson et al., 2013; Abdel-Moein and Hamza, 2017), and have the potential to cause query (Q) fever (Musso et al., 2014). The Q-fever was reported for the first time in 1935 from Australia as an outbreak of febrile illness with flu-like symptoms (Derrick, 1937), and its causative agent was initially named *Rickettsia burnetii*, but later on renamed as *C. burnetii* (Philip, 1948). *Coxiella* spp. have been isolated from almost 40 tick species, and hence considered as its tick-borne transmission to animals and humans (Eklund et al., 1947; Beaman and Hung, 1989; Duron et al., 2014). Tick species, including *Hyalomma dromedarii, Hyalomma anatolicum, Hyalomma scupense, Rhipicephalus microplus*, and *Rhipicephalus annulatus*, may serve as vector reservoirs for the transmission of *C. burnetii* in Pakistan (Karim et al., 2017). *Coxiella*-like endosymbionts were also found in various tick species, and an obligatory mutualism between this bacteria and host ticks has been proven (Smith et al., 2015).

Ticks can transmit *Coxiella* spp. both transovarially and transstadially to their offspring. Infected ticks excrete enormous amounts of *Coxiella* spp. in their feces, contaminating the skin of host animals and playing a significant role in the spread of *Coxiella* infection (Cong et al., 2015; Seo et al., 2016). *Coxiella burnetii* may resist harsh environmental factors, for instance, dry and hot weather, desiccation, and other antiseptics. As it may affect the productive and reproductive abilities, humans and animals could face long-term infection risks (Ullah et al., 2019). Various techniques have been effectively followed for the surveillance of *C. burnetii* infection. Still, ELISA is considered an effective technique for its serological diagnosis. In combination with sequencing, PCR is believed to be the best technique for the molecular identification and genetic characterization of *C. burnetii* (Niemczuk et al., 2014; Bontje et al., 2016).

*Coxiella* spp. have been detected in different tick species, animals, humans, and soil samples in Asia that have been reported in various studies. *Coxiella burnetii* is the causative agent of Q-fever, one of the ignored zoonoses in developing countries, including Pakistan. To the best of our knowledge, approximately 24 studies from 1955 to 2022 regarding this infection have been reported in Pakistan, and Q-fever in humans, goats, sheep, cattle, buffaloes, as well as rodents has been serologically documented (Ahmed, 1987; Ullah et al., 2019; Ali et al., 2022a; Hussain et al., 2022). The *C. burnetii* is also considered a soil-borne pathogen as its isolation has been confirmed from soil samples (Shabbir et al., 2015). Due to the information dearth regarding numerous tick-borne pathogens (TBPs) that infect ruminants and other animals in Pakistan, substantial research is required to investigate the genetic composition of various TBPs, specifically *Coxiella* spp. Hence, this study aimed to molecularly characterize different tick species infesting domestic and wild animals and screen out the associated *Coxiella* spp. in Pakistan and summarize the association of *Coxiella* spp. with ticks infesting various hosts in Asia.

## Materials and methods

### Ethical considerations

The Advance Studies and Research Board (ASRB: Dir/A&R/AWKUM/2022/9396) of the Department of Zoology, Abdul Wali Khan University Mardan, Pakistan, approved prior consent for this study. Additionally, permission was taken from the owners of the animals to observe hosts and ticks collection. All the rules regarding animal welfare regulations were followed while handling the animals.

### Description of the study area and sampling sites

**Figure 1:**
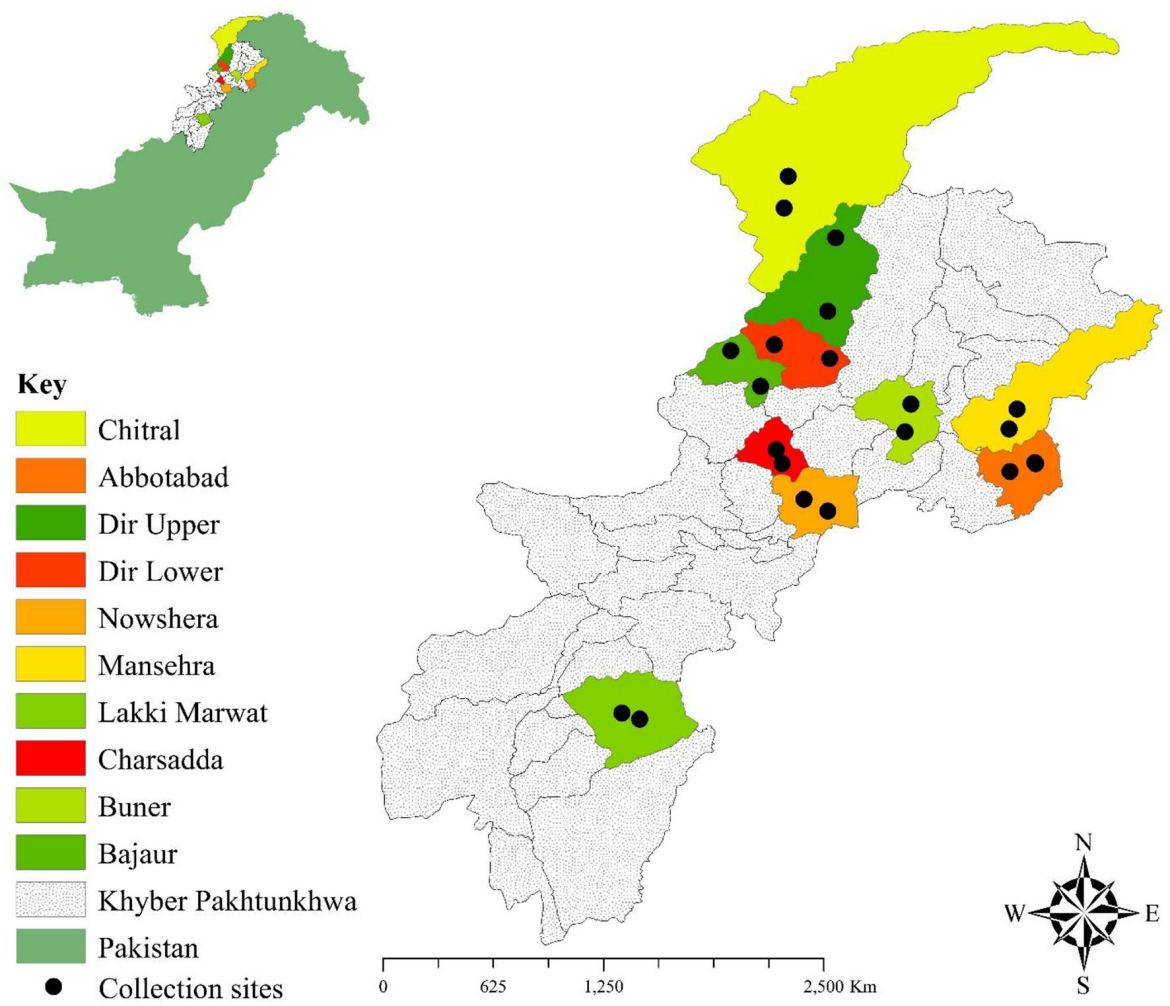
Map showing the collection sites where ticks were collected.

Different districts including Lakki Marwat (32.5993° N, 70.9160° E), Mansehra (34.3271° N, 73.1992° E), Bajaur (34.7522° N, 71.5162° E), Dir Upper (35.3274° N, 72.0907° E), Dir Lower (34.8364° N, 71.8964° E), Abbottabad (34.1534° N, 73.2215° E), Buner (34.459129° N, 72.557252° E), Charsadda (34.161297° N, 71.755377° E), Chitral (35.727064° N, 71.759794° E), and Nowshera (33.998608° N, 71.999144° E) of Khyber Pakhtunkhwa were selected for the current study. These study locations have desertic plains, arid plains, arid hilly, humid plains, and hilly areas with variations in their climatic conditions, altitude, and seasons (winter, spring, summer, and autumn). The summer season is comparatively hot and longer in district Lakki Marwat than in other districts; however, snowfall occurs in winter in the districts of Chitral, Mansehra, Buner, Bajaur, Dir Upper, Dir Lower, and Abbottabad (climate-data.org; accessed on 20 February 2023). Goats, sheep, camels, and cattle are the livestock of the region that are intended for producing dairy products and transportation. These transhumant animals move from one place to another within the district for food and natural pastures. Their diet and resources depend upon climatic conditions that remarkably vary spatiotemporally. The Global Positioning System was used for the geographical coordinates of the districts as mentioned above and designed the study map through ArcGIS v 10.3.1 (Figure 1).

### Tick collection and their morphological identification

The herds of goats, sheep, camels, and cattle were visited for tick collection from September 2021 to August 2022. Moreover, wild mice captured by local farmers on agricultural land and domestic fowls (*Gallus gallus domesticus*) were also examined for tick specimens. Tick specimens were collected manually from different hosts in the study districts. The collected ticks were morphologically identified under a stereomicroscope (SZ61, Olympus, Japan) using available standard morphological keys (Hoogstraal and Kaiser, 1959; Hoogstraal and Varma, 1962; Hoogstraal and Trapido, 1966; Dhanda and Kulkarni, 1969; Kohls et al., 1970; Cerný and Hoogstraal, 1977; Apanaskevich, 2003; Apanaskevich et al., 2008; Ahmad et al., 2022; Ali et al., 2022b). The identified tick species were categorized according to species, gender, and nymph stage or adult stage, and then preserved in 100% ethanol at room temperature before further analyses.

### DNA extraction and molecular screening

The preserved ticks were washed with 70% ethanol, followed by their immersion in distilled water for 10 min to eliminate the external contamination, and subsequently dried on a sterile filter paper. A subset of 227 (137 N, 49 F, and 41 M) ticks including 65 *Ha*. *cornupunctata* (41 N, 14 F, and 10 M), 28 *Ha. montgomeryi* (17 N, 6 F, and 5 M), 28 *Hy. anatolicum* (17 N, 6 F, and 5 M), 27 *A. persicus* (17 N, 6 F, and 4 M), 23 *R. haemaphysaloides* (15 N, 5 F, and 3 M), 19 *R. microplus* (9 N, 5 F, and 5 M), 19 *Ha. bispinosa* (11 N, 4 F, and 4 M), 12 *Hy. dromedarii* (7 N, 1 F, and 4 M), and 6 *Ha. danieli* (3 N, 2 F, and 1 M) were randomly selected and used individually for DNA extraction. Each stage of the morphologically identified tick species was individually crushed using sterile scissors to extract genomic DNA through the standard protocol of the phenol–chloroform method (Sambrook et al., 1989).

The whole extracted genomic DNA of each morphologically identified tick species (each stage) was individually used to amplify *cox1* fragments by utilizing species-specific primers in a conventional PCR (Table 1). Nested PCR was performed to amplify the *GroEL* fragment of *Coxiella* spp. (GE-96G, BIOER, Hangzhou, China). In nested PCR, two pairs of primers were used for the said purpose (Table 1). PCR reaction mixtures were performed in 25 μL, comprised of 1 μL of each primer at a concentration of 10 pmol/μL (first pair of primers in case of *GroEL*), 8.5 μL PCR water, 2 μL (100 ng/μL) genomic DNA, and 12.5 μL Dream*Taq* MasterMix (2 × ) (Thermo Fisher Scientific, Inc., Waltham, MA, USA). However, 2 μL of PCR product from the first PCR amplified reaction was used instead of genomic DNA in the second PCR run (in the case of *GroEL*) along with the second pair of primers at the same concentration. In each PCR reaction, PCR water was taken as a negative control, while *Hyalomma scupense* and *Rickettsia massiliae* DNA were taken as a positive control for ticks and *Coxiella*, respectively. The amplified products were loaded in 2% agarose gel to observe the expected band through the Gel Documentation System (BioDoc-It™ Imaging Systems, UVP, LLC, Upland, CA, USA). PCR amplified products were purified *via* GeneClean II Kit (Qbiogene, Il-lkirch, France) following the manufacturer's protocol. The amplified amplicons were sequenced bidirectionally through the Sanger-based sequencing method (Macrogen, Inc., Seoul, South Korea).

**Table 1:** Primers and PCR cycling conditions used in the current study.

**Gene**	**Sequence (5-3)**	**Amplicon size**	**Cycling conditions**	**References**
***cox1***	HCO2198: TAAACTTCAGGGTGACCAAAAAATCA	710 bp	$\frac{98^{•}C}{30\;sec}\to 40X\left[\;\frac{98^{•}C}{10\;sec}\;\to \frac{63^{•}C}{20\;sec}\to \frac{72^{•}C}{25\;sec}\right]\to \frac{72^{•}C}{5\;min}\to 10^{•}C$	Folmer et al., 1994
	LCO1490: GGTCAACAAATCATAAAGATATTGG			
***GroEL***	CoxGrF1: ^*^ TTTGAAAAYATGGGCGCKCAAATGGT	655 bp	$\frac{95^{•}C}{3\;min}\to 30X\left[\;\frac{95^{•}C}{30\;sec}\to \frac{56^{•}C}{30\;sec}\to \frac{72^{•}C}{1.5\;min}\right]\to \frac{72^{•}C}{7\;min}\to 10^{•}C$	Duron et al., 2014
	CoxGrR2: ^*^ CGRTCRCCAAARCCAGGTGC			
	CoxGrF2: ^**^ GAAGTGGCTTCGCRTACWTCAGACG	619 bp		
	CoxGrFR1: ^**^ CCAAARCCAGGTGCTTTYAC			

* First run of nested PCR.

** Second run of nested PCR.

### Sequences and phylogenetic analyses

The chromatograms of all the obtained sequences were manually observed and trimmed for purification purposes to remove the contaminated and poor reading regions through SeqMan V. 5 (DNASTAR, Inc., Madison, WI, USA). Final trimmed sequences were subjected to Basic Local Alignment Search Tool (BLAST) (Altschul et al., 1990) at National Center for Biotechnology Information to get the high identity sequences in FASTA format. ClustalW multiple alignments (Thompson et al., 1994) were used to align all the downloaded sequences along with the obtained and selected outgroup sequences in BioEdit Sequence Alignment Editor V.7.0.5 (Raleigh, NC, USA) (Hall et al., 2011). The phylogenetic trees based on partial fragments of *cox1* and *GroEL* were constructed in MEGA-X (Molecular Evolutionary Genetics Analysis) (Kumar et al., 2018) through the neighbor-joining method (Tamura-Nei model) and the Maximum Parsimony method (Tamura-Nei model) (Tamura and Nei, 1993) with support of 1000 bootstrapping replicons, respectively. The coding fragments (*cox1* and *GroEL*) were aligned using MUSCLE (Edgar, 2004).

### Literature search

The literature search was conducted using databases such as PubMed, Google Scholar, and Web of Sciences, to overview the published studies regarding the detection of *C. burnetii* in different ticks, animals, humans, or soil in Asia. The keywords used for the search were as follows: tick(s), small ruminant(s), livestock, *C. burnetii*, Coxiellosis, and Q-fever. Combinations of the aforementioned various keywords were used to retrieve full-text research articles, review articles, short communications, and conference papers. Reference lists of retrieved articles were screened to identify relevant articles (accessed on 16 April 2023) (Table 2).

**Table 2:** *Coxiella burnetii* detected in different ticks, animals, humans, or soil samples in Asia.

**Country/year**	**Tick spp./source**	**Detected/infested host**	**Serologically/molecularly**	**Reference**
Abu Dhabi/2021	Blood	Camels	Serologically	El Tigani-Asil et al., 2021
Afghanistan/2012–2013	Blood	Humans	Serologically	Akbarian et al., 2015
		Livestock		
Armenia/1971-1974	Blood	Humans	Serologically	Tarasevič et al., 1976
		Cattle		
Azerbaijan/2018	Milk	Goats, sheep	Molecularly (PCR)	Khademi et al., 2020
Bangladesh/2018-2021	Blood	Cattle and goats, Humans	Serologically	Chakrabartty et al., 2021
	Milk samples	Cattle	Molecularly (PCR)	
Bhutan/2014-2015	Blood	Humans (patients)	Serologically and Molecularly (PCR)	Tshokey et al., 2018
Bhutan/2015		Goats	Serologically	Tshokey et al., 2019
Bangladesh/2007-2008	Blood	Cattle, goats, sheep	Serologically	Rahman et al., 2016
	Placenta	Sheep	Molecularly (PCR)	
Cambodia/2019	Blood	Goats	Serologically	Siengsanan-Lamont et al., 2023
China	Blood	Humans	Serologically and Molecularly (PCR)	El-Mahallawy et al., 2016
China/2018	Tissue (spleens)	Hedgehogs	Molecularly (PCR and sequencing)	Gong et al., 2020
China/2018-2019	*D. nuttalli*	Cattle, sheep	Molecularly (PCR and sequencing)	Ni et al., 2020
	*D. pavlovskyi*			
	*D. silvarum*			
	*D. niveus*			
	*Hy. rufipes*			
	*Hy. anatolicum*			
	*Hy. asiaticum*			
	*R. sanguineus*			
	*Ha. punctata*			
Cyprus	Blood	Humans	Serologically	Psaroulaki et al., 2006
		Goats and sheep		
	*R. sanguineus* and *Hyalomma* spp.	Goats and sheep	Molecularly (PCR)	
Hong Kong/2008	Blood	Humans	Serologically	Chan et al., 2010
India	Blood	Humans	Serologically	Sahu et al., 2018
India/2018-2019	Blood	Goats	Serologically and Molecularly (PCR and sequencing)	Patra et al., 2020
	*R. microplus*			
Iran/2013-2016	Blood	Humans	Molecularly (PCR)	Esmaeili et al., 2019
Iran/2017–2018	Aborted samples	Cattle, sheep, and goats	Molecularly (PCR)	Mohabati Mobarez et al., 2021
Spleen, liver, and cotyledons
Iraq/2019	Blood	Camels	Serologically and Molecularly (PCR and sequencing)	Al-Graibawi et al., 2021
Iraq/2007	Topsoil and airborne dust		Molecularly (PCR)	Leski et al., 2011
Iraq/2018-2019	Blood and milk	Cows	Serologically and Molecularly (PCR and sequencing)	Gharban and Yousif, 2020
Iraq/2005	Blood	Humans (US military)	Serologically	Royal et al., 2013
Israel/2005	Blood	Humans	Serologically and Molecularly (PCR)	Amitai et al., 2010
Israel	*Hy. dromedarii*	Camels	Molecularly (PCR)	Mumcuoglu et al., 2022
	*Hy. aegyptium*	Tortoises		
Indonesia/2017	Tissue	Cows, goats, sheep	Molecularly (PCR and sequencing)	Rini et al., 2022
Japan/1996–1997	Blood	Humans	Molecularly (PCR)	Kato et al., 1998
Jordan/2015–2016	Blood	Humans	Serologically	Obaidat et al., 2019
Jordan/2015–2017	Blood	Goats, sheep	Serologically	Lafi et al., 2020
Kazakhstan/2021–2022	*D. marginatus* and *Hy. anatolicum*	Cattle	Molecularly (PCR and sequencing)	Sultankulova et al., 2022
Korea/2016	Blood	Humans	Molecularly (PCR and sequencing)	Lee et al., 2020
Kuwait/2007	Topsoil and airborne dust		Molecularly (PCR)	Leski et al., 2011
Lebanon/2015	Blood	Humans	Serologically	Dabaja et al., 2018
Lebanon/2014	*R. annulatus*	Cattle, sheep, goats	Molecularly (PCR)	Dabaja et al., 2020
	*R. turanicus*			
	*Hy. anatolicum*			
	*R. sanguineus*			
	*R. bursa*			
	Milk	Serologically		
Laos/2016–2017	Blood	Goats	Serologically	Burns et al., 2018
Malaysia/2012–2013	Blood	Humans	Serologically	Khor et al., 2018
Malaysia/2013	Blood and vaginal sample	Cattle	Molecularly (PCR and sequencing)	Nurkunasegran et al., 2017
	*Amblyomma* and *Dermacentor* spp.	Rodents		
	*Haemaphysalis* spp.	Vegetation		
	*R. sanguineus* and *Dermacentor* spp.	Dogs		
Mongolia/2008–2015	Blood	Snow leopards	Serologically	Esson et al., 2019
	Ticks		Molecularly (PCR)	
Nepal/2016	Blood	Cattle	Serologically	Panth et al., 2017
Oman/2019	Blood/bone	Humans	Serologically	Al-Kindi et al., 2022
Pakistan	Blood	Humans, goats, sheep, buffaloes, cows, Rodents	Serologically	Ahmed, 1987
Pakistan	Soil	Soil	Molecularly (PCR)	Shabbir et al., 2015
Pakistan	Blood	Camels	Serologically	Hussain et al., 2022
Pakistan/2016	Ticks and blood	Goats and sheep	Serologically and Molecularly (PCR)	Ullah et al., 2019
Pakistan	Blood	Humans	Serologically	Ali et al., 2022a
Palestine/2016	Blood	Rams	Serologically	Jalboush and Alzuheir, 2017
Qatar/2005–2006	Blood	Humans (US military)	Serologically	Royal et al., 2013
Saudi Arabia	Blood, milk, feces, and urine	Camels, cattle, and goats	Molecularly (PCR)	Mohammed et al., 2014
Saudi Arabia/2011–2013	Blood	Humans	Serologically	Almogren et al., 2013
South Korea/2007–2013	Blood	Horses	Serologically	Seo et al., 2016
Taiwan/2007	Blood	Humans	Serologically	Chang et al., 2010
Taiwan/2009–2011	Blood	Dogs	Molecularly (PCR and sequencing)	Chou et al., 2014
Thailand/2012–2013	Blood	Humans	Serologically and Molecularly (PCR)	Doung-Ngern et al., 2017
		Cattle		
	Milk	Cattle		
Turkey	Blood	Cows, sheep, goats	Serologically	Ozgen et al., 2022
Turkey/2007	Blood	Humans	Serologically	Kilic et al., 2008
Tunisia/2015–2017	*Hy. impeltatum*	Camels	Molecularly (PCR and sequencing)	Selmi et al., 2019
	*Hy. dromedarii*			
United Arab Emirates	Blood	Goats, sheep	Serologically	Barigye et al., 2022
Vietnam	Bone marrow	Humans (patient)	Molecularly (PCR)	Thi Vinh An et al., 2022

## Results

### Hosts prevalence

The highest number of observed hosts (374/678, 55.2%) were included in a group having age > 3 years, followed by various hosts (192/678, 28.3%) having age 1–3 years, and the lowest numbers of observed hosts (112/678, 16.5%) belonging to the age group having age < 1 year. Different hosts having age >3 years were highly infested (192/345, 55.6%), while animals having age ≤ 1 year were least infested (62/345, 18.0%). The examined and infested female hosts were more predominant in number (486/678; 71.7%, 237/345; 68.7%) than male hosts (192/678; 28.3%, 108/345; 31.3%). The highest number of infested hosts was recorded in summer (June–August) (146/345, 42.3%), followed by spring (March–May) (99/345, 28.7%), autumn (September–November) (65/345, 18.8%), and winter (December–February) (35/345, 10.4%), respectively (Table 3).

**Table 3:** Age, gender, and season-wise infestation rate of hosts.

**Variables**		**Observed host (%)**	**Infested hosts (%)**	**Total infested/total observed (%)**
Age	< 1 year	112 (16.5)	62 (18.0)	345/678 (52.1)
	1–3 years	192 (28.3)	91 (26.4)	
	>3 years	374 (55.2)	192 (55.6)	
Gender	Female	486 (71.7)	237 (68.7)	345/678 (52.1)
	Male	192 (28.3)	108 (31.3)	
Seasons	Spring (March–May)	163 (24.0)	99 (28.7)	345/678 (52.1)
	Summer (June–August)	191 (28.2)	146 (42.3)	
	Autumn (September–November)	165 (24.3)	65 (18.8)	
	Winter (December–February)	159 (23.4)	35 (10.1)	

A total of 678 different hosts such as goats, sheep, camels, cattle, wild mice, and domestic fowls were examined for tick collection in the selected localities, of which other hosts (number = 345/678, 50.9%), including goats (78/149, 52.3%), sheep (75/136, 55.1%), camels (36/93, 38.7%), cattle (69/129, 53.5%), wild mice (15/48, 31.2%), and domestic fowls (72/123, 58.5%) were found tick infested. The highest prevalence of infested hosts was recorded in district Lakki Marwat (44/351, 12.5%), followed by Charsadda (36/351, 10.3%), Nowshera, Buner, Mansehra, and Abbottabad (35/351, 9.7%), Chitral (34/351, 9.7%), Dir Upper and Dir Lower (33/351, 9.4%), while least infestation rate was recorded in district Bajaur (31/351, 8.8%) (Table 4).

**Table 4:** Occurrence of ticks and molecular detection of *Coxiella* spp. in different districts of Khyber Pakhtunkhwa.

**Districts**	**Hosts**			**Tick species**	**Ticks life stages**	**Molecularly analyzed (DNA extraction and PCR)**	**Molecularly screened** ***Coxiella*** **spp**.							
	**Animal type**	**Observed**	**Infested**		**(N, F, M) Total**	**(N, F, M) Total**	***Coxiella burnetii*** **(OQ883856)**	***Coxiella*** **endosymbiont (OQ883857)**	***Coxiella*** **endosymbiont (OQ883858)**	***Coxiella*** **sp. (OQ883859)**	***Coxiella*** **sp. (OQ883860)**	***Coxiella*** **sp. (OQ883861)**	***Coxiella*** **sp. (OQ883862)**	***Coxiella*** **sp. (OQ883863)**
Lakki Marwat	Goats	19	8	*R. haemaphysaloides*	(5 N, 3 F, 1 M) 9	(1 N, 1 F) 2	–	–	–	–	–	–	–	–
				*Hy. anatolicum*	(7 N, 3 F, 2 M) 12	(1 N, 1 M) 2	–	–	–	–	–	–	–	–
				*Ha. bispinosa*	(6 N, 2 F, 3 M) 11	(1 N, 1 M) 2	–	–	–	–	–	–	–	–
	Sheep	18	7	*R. haemaphysaloides*	(5 N, 2 F, 1 M) 8	(2 N, 1 F) 3	–	–	–	–	–	–	–	–
				*Ha. montgomeryi*	(7 N, 2 F, 3 M) 12	(1 N, 1 M) 2	–	–	–	–	–	–	–	–
				*Ha. cornupunctata*	(5 N, 1 F, 3 M) 9	(1 N) 1	–	–	–	–	–	–	–	–
	Camels	31	13	*Hy. dromedarii*	(6 N, 3 F, 3 M) 12	(3 N, 1 F, 2 M) 6	2 N, 1 F	–	–	–	–	–	–	–
				*Hy. anatolicum*	(8 N, 4 F, 3 M) 15	(2 N, 1 F, 1 M) 4	1 N, 1 F	–	–	–	–	–	–	–
	Cattle	17	8	*R. microplus*	(10 N, 3 F, 4 M) 17	(1 N, 1 M) 2	–	–	–	–	–	–	–	–
				*Hy. anatolicum*	(6 N, 4 F, 1 M) 11	(1 N) 1	–	–	–	–	–	–	–	–
	Wild mice	4	2	*Ha. cornupunctata*	(4 N, 3 F) 7	(2 N, 1 F) 3	–	–	–	–	–	–	–	–
	Domestic fowls	17	6	*A. persicus*	(8 N, 4 F, 2 M) 14	(2 N, 1 F, 1 M) 4	–	–	–
Charsadda	Goats	17	7	*Ha. cornupunctata*	(5 N, 3 F, 1 M) 9	(1 N, 1 F) 2	–	–	–	–	–	–	–	–
				*Hy. anatolicum*	(6 N, 3 F, 3 M) 12	(1 N, 1 M) 2	–	–	–	–	–	–	–	–
				*Ha. bispinosa*	(5 N, 2 F, 2 M) 9	(1 N, 1 F, 1 M) 3	–	–	–	–	–	–	–	–
	Sheep	15	5	*R. haemaphysaloides*	(4 N, 2 F, 2 M) 8	(1 N) 1	–	–	–	–	–	–	–	–
				*Ha. montgomeryi*	(7 N, 3 F, 2 M) 12	(1 N, 1 F, 1 M) 3	–	–	–	–	–	–	–	–
				*Ha. cornupunctata*	(5 N, 2 F, 2 M) 9	(1 N, 1 F) 2	–	–	–	–	–	–	–	–
	Camels	26	8	*Hy. dromedarii*	(7 N, 3 F, 2 M) 12	(1 N, 1 M) 2	–	–	–	–	–	–	–	–
				*Hy. anatolicum*	(5 N, 4 F, 2 M) 11	(1 N) 1	–	–	–	–	–	–	–	–
	Cattle	15	7	*R. microplus*	(10 N, 6 F, 1 M) 17	(1 N, 1 F, 1 M) 3	–	–	–	–	–	–	–	–
				*Hy. anatolicum*	(8 N, 4 F, 3 M) 15	(1 N, 1 F) 2	–	–	–	–	–	–	–	–
	Wild mice	11	3	*Ha. cornupunctata*	(4 N, 3 M) 7	(4 N, 1 F) 5	3 N, 1 F	–	–	–	–	–	–	–
	Domestic fowls	13	6	*A. persicus*	(9 N, 4 F, 3 M) 16	(1 N, 1 M) 2	–	–	–	–	–	–	–	–
Nowshera	Goats	19	7	*Ha. bispinosa*	(5 N, 3 F, 1 M) 9	(1 N, 1 F) 2	–	–	–	–	–	–	–	–
				*Ha. cornupunctata*	(6 N, 3 F, 3 M) 12	(1 N, 1 F, 1 M) 3	–	–	–	–	–	–	–	–
	Sheep	11	6	*R. haemaphysaloides*	(5 N, 4 F, 1 M) 10	(1 N) 1	–	–	–	–	–	–	–	–
				*Ha. montgomeryi*	(4 N, 2 F, 2 M) 8	(2 N) 2	–	–	–	–	–	–	–	–
	Camels	21	8	*Hy. dromedarii*	(7 N, 2 F, 3 M) 12	(1 N, 1 M) 2	–	–	–	–	–	–	–	–
	Cattle	13	6	*R. microplus*	(8 N, 4 F, 2 M) 14	(1 N, 1 F) 2	–	–	–	–	–	–	–	–
	Wild mice	3	1	*Ha. cornupunctata*	(4 N, 1 F, 1 M) 6	(1 N, 1 F) 2	–	–	–	–	–	–	–	–
	Domestic fowls	15	7	*A. persicus*	(9 N, 2 F, 3 M) 14	(2 N, 1 F) 3	–	–	–	–	–	–	–	–
Dir Upper	Goats	16	9	*Ha. cornupunctata*	(7 N, 3 F, 3 M) 13	(1 N, 1 F, 1 M) 3	–	–	–	–	–	–	–	–
				*Ha. bispinosa*	(6 N, 2 F, 3 M) 11	(1 N, 1 M) 2	–	–	–	–	–	–	–	1 N, 1 M
				*Ha. danieli*	(8 N, 4 F, 2 M) 14	(3 N, 2 F, 1 M) 6	–	–	–	–	–	–	–	–
	Sheep	15	7	*R. haemaphysaloides*	(5 N, 3 F, 2 M) 10	(1 N, 1 F) 2	–	–	–	–	–	–	–	–
				*Ha. montgomeryi*	(6 N, 2 F, 1 M) 9	(1 N, 1 F) 2	–	–	–	–	–	–	–	–
				*Ha. cornupunctata*	(7 N, 3 F, 2 M) 12	(1 N) 1	–	–	–	–	–	–	–	–
	Cattle	12	10	*R. microplus*	(9 N, 2 F, 4 M) 15	(1 N, 1 M) 2	–	–	–	–	–	–	–	–
	Wild mice	2	1	*Ha. cornupunctata*	(4 N, 2 F, 2 M) 8	(1 N) 1	–	–	–	–	–	–	–	–
	Domestic fowls	10	6	*A. persicus*	(7 N, 3 F, 2 M) 12	(1 N) 1	–	–	–	–	–	–	–	–
Dir Lower	Goats	13	10	*Ha. cornupunctata*	(5 N, 2 F, 2 M) 9	(5 N, 2 F, 1 M) 8	1 N, 1 F	2 N, 1 F	–	1 N, 1 M	–	–	–	–
				*Ha. bispinosa*	(6 N, 3 F, 3 M) 12	(1 N) 1	–	–	–	–	–	–	–	–
	Sheep	14	9	*Ha. montgomeryi*	(4 N, 2 F, 2 M) 8	(1 N) 1	–	–	–	–	–	–	–	–
				*Ha. cornupunctata*	(8 N, 3 F, 3 M) 14	(2 N, 1 F, 2 M) 5	1 N, 1 F, 2 M	–	–	–	–	–	–	–
	Cattle	13	5	*R. microplus*	(7 N, 4 F, 2 M) 13	(1 N) 1	–	–	–	–	–	–	–	–
				*Hy. anatolicum*	(9 N, 4 F, 2 M) 15	(1 N, 1 F) 2	–	–	–	–	–	–	–	–
	Wild mice	5	1	*Ha. cornupunctata*	(5 N, 5 F) 10	(1 N, 1 F) 2	–	–	–	–	–	–	–	–
	Domestic fowls	11	8	*A. persicus*	(8 N, 3 F, 3 M) 14	(1 N, 1 M) 2	–	–	–	–	–	–	–	–
Buner	Goats	14	7	*Ha. cornupunctata*	(5 N, 4 F, 2 M) 11	(2 N) 2	–	–	–	–	–	–	–	–
				*Hy. anatolicum*	(8 N, 3 F, 5 M) 16	(1 N) 1	–	–	–	–	–	–	–	–
				*Ha. bispinosa*	(6 N, 4 F) 10	(1 N) 1	–	–	–	–	–	–	–	–
	Sheep	13	10	*R. haemaphysaloides*	(5 N, 2 F, 2 M) 9	(1 N, 1 F) 2	–	–	–	–	–	–	–	–
				*Ha. montgomeryi*	(6 N, 5 F, 1 M) 12	(2 N, 1 F) 3	–	–	2 N, 1 F	–	–	–	–	–
				*Ha. cornupunctata*	(5 N, 4 F, 1 M) 10	(1 N) 1	–	–	–	–	–	–	–	–
	Cattle	11	9	*R. microplus*	(8 N, 4 F, 3 M) 15	(1 N, 1 F) 2	–	–	–	–	–	–	–	–
				*Hy. anatolicum*	(7 N, 3 F, 3 M) 13	(1 N, 1 M) 2	–	–	–	–	–	–	–	–
	Wild mice	3	1	*Ha. cornupunctata*	(5 N, 3 F, 1 M) 9	(1 N) 1	–	–	–	–	–	–	–	–
	Domestic fowls	13	8	*A. persicus*	(6 N, 5 F, 2 M) 13	(1 N, 1 F) 2	–	–	–	–	–	–	–	–
Mansehra	Goats	12	8	*Ha. cornupunctata*	(5 N, 2 F, 1 M) 8	(2 N, 1 F) 3	–	–	–	–	–	–	–	–
				*Hy. anatolicum*	(7 N, 3 F, 4 M) 14	(1 N) 1	–	–	–	–	–	–	–	–
	Sheep	10	9	*R. haemaphysaloides*	(6 N, 2 F, 3 M) 11	(3 N, 1 F, 2 M) 6	–	2 N, 1 F, 1 M	–	–	–	–	–	–
				*Ha. montgomeryi*	(4 N, 3 F) 7	(1 N, 1 F) 2	–	–	–	–	–	–	–	–
				*Ha. cornupunctata*	(6 N, 2 F, 2 M) 10	(2 N) 2	–	–	–	–	–	–	–	–
	Cattle	14	9	*R. microplus*	(9 N, 4 F, 3 M) 16	(1 N, 1 F) 2	–	–	–	–	–	–	–	–
				*Hy. anatolicum*	(7 N, 3 F, 4 M) 14	(1 N, 1 M) 2	–	–	–	–	–	–	–	–
	Wild mice	5	2	*Ha. cornupunctata*	(5 N, 2 F, 3 M) 10	(1 N) 1	–	–	–	–	–	–	–	–
	Domestic fowls	9	7	*A. persicus*	(9 N, 3 F, 4 M) 16	(2 N, 1 F) 3	–	–	–	–	–	–	–	–
Bajaur	Goats	16	6	*Ha. bispinosa*	(5 N, 4 F, 3 M) 12	(1 N) 1	–	–	–	–	–	–	–	–
				*Ha. montgomeryi*	(9 N, 3 F, 4 M) 16	(3 N, 1 F, 1 M) 5	–	–	–	–	–	–	–	–
				*Ha. cornupunctata*	(4 N, 5 F) 9	(2 N, 1 F) 3	–	–	–	1 F	–	–	–	–
	Sheep	17	7	*R. haemaphysaloides*	(6 N, 3 F, 3 M) 12	(2 N) 2	–	–	–	–	–	–	–	–
				*Ha. montgomeryi*	(8 N, 2 F, 4 M) 14	(3 N, 1 F, 1 M) 5	–	–	–	–	2 N	1 F	–	–
	Cattle	12	8	*Hy. anatolicum*	(7 N, 4 F, 3 M) 14	(1 N) 1	–	–	–	–	–	–	–	–
	Wild mice	8	1	*Ha. cornupunctata*	(5 N, 4 F, 1 M) 10	(1 N, 1 M) 2	–	–	–	–	–	–	–	–
	Domestic fowls	14	9	*A. persicus*	(8 N, 3 F, 3 M) 14	(1 N) 1	–	–	–	–	–	–	–	–
Abbottabad	Goats	13	9	*Ha. cornupunctata*	(6 N, 2 F, 4 M) 12	(1 N, 1 F, 1 M) 3	–	–	–	–	–	–	–	–
				*Hy. anatolicum*	(5 N, 2 F, 3 M) 10	(1 N) 1	–	–	–	–	–	–	–	–
				*Ha. bispinosa*	(4N, 3 F, 1M) 8	(3N, 1 F, 1M) 5	–	–	–	–	–	–	2N,1F	–
	Sheep	12	6	*R. haemaphysaloides*	(7 N, 2 F, 3 M) 12	(1 N, 1 M) 2	–	–	–	–	–	–	–	–
				*Ha. montgomeryi*	(5 N, 3 F, 1 M) 9	(1 N) 1	–	–	–	–	–	–	–	–
				*Ha. cornupunctata*	(6 N, 4 F, 2 M) 12	(1 N, 1 M) 2	–	–	–	–	–	–	–	–
	Camels	15	7	*Hy. dromedarii*	(6 N, 4 F, 1 M) 11	(2 N) 2	–	–	–	–	–	–	–	–
				*Hy. anatolicum*	(9 N, 5 F, 14	(1 N, 1 F) 2	–	–	–	–	–	–	–	–
	Cattle	9	7	*R. microplus*	(9 N, 4 F, 3 M) 16	(1 N, 1 M) 2	–	–	–	–	–	–	–	–
	Wild mice	3	1	*Ha. cornupunctata*	(5 N, 2 M) 7	(2 N, 1 M) 3	–	–	–	–	–	–	–	–
	Domestic fowls	9	5	*A. persicus*	(8 N, 4 F, 3 M) 15	(1 N, 1 F) 2	–	–	–	–	–	–	–	–
Chitral	Goats	10	7	*Ha. cornupunctata*	(5 N, 2 F, 2 M) 9	(1 N, 1 M) 2	–	–	–	–	–	–	–	–
				*Hy. anatolicum*	(8 N, 3 F, 1 M) 12	(1 N, 1 F) 2	–	–	–	–	–	–	–	–
				*Ha. bispinosa*	(6 N, 2 F, 2 M) 10	(1 N, 1 F) 2	–	–	–	–	–	–	–	–
	Sheep	11	9	*R. haemaphysaloides*	(5 N, 3 F) 8	(2 N) 2	–	–	–	–	–	–	–	–
				*Ha. montgomeryi*	(7 N, 3 F, 2 M) 12	(1 N, 1 M) 2	–	–	–	–	–	–	–	–
				*Ha. cornupunctata*	(5 N, 3 F, 1 M) 9	(1 N) 1	–	–	–	–	–	–	–	–
	Cattle	13	6	*Hy. anatolicum*	(5 N, 3 F, 3 M) 11	(1 N, 1 F) 2	–	–	–	–	–	–	–	–
				*R. microplus*	(7 N, 4 F, 3 M) 14	(1 N, 1 F, 1 M) 3	–	–	–	–	–	–	–	–
	Wild mice	4	2	*Ha. cornupunctata*	(4 N, 2 F, 1 M) 7	(1 N) 1	–	–	–	–	–	–	–	–
	Domestic fowls	12	10	*A. persicus*	(9 N, 5 F, 4 M) 18	(5 N, 1 F, 1 M) 7	–	2 N, 1 F, 1 M	–	–	–	–	–	–
Total	**678**	**345** (50.9%)	(613 N, 293 F, 213 M) **1,119**	(137 N, 49 F, 41 M) **227**	**15** (6.7%)	**14** (6.3%)	**11** (4.9%)					

### Ticks and their molecular analyses

Altogether, 1,119 ticks including nymphs (613/1,119, 54.8%), adult females (293/1,119, 26.2%), and males (213/1,119, 19.0%) were collected from infested hosts and were categorized into four genera (*Haemaphysalis, Hyalomma, Rhipicephalus*, and *Argas*). The highest number of collected tick species was *Ha. cornupunctata* (258/1,119, 23.1%), followed by *Hy. anatolicum* (209/1,119, 18.7%), *A. persicus* (146/1,119, 13.0%), *R. microplus* (137/1,119, 12.2%), *Ha. montgomeryi* (119/1,119, 10.6%), *R. haemaphysaloides* (97/1,119, 8.7%), *Ha. bispinosa* (92/1,119, 8.2%), *Hy. dromedarii* (47/1,119, 4.2%), and *Ha. danieli* (14/1,119, 1.2%). Genomic DNA was extracted from 227 morphologically identified ticks (137 N, 49 F, and 41 M), and all identified tick species were molecularly confirmed *via* sequencing of the *cox1* partial fragment (Table 4).

### Screening of *Coxiella* in various ticks

Extracted DNA of 227 (20.3%) was used to amplify the fragments of *GroEL* of *Coxiella* spp. A total of 40/227 (17.6%) ticks were found positive for *Coxiella* spp., including *C. burnetii* (15, 6.7%), *Coxiella* endosymbionts (14, 6.3%), and *Coxiella* sp. (11, 4.9%). *Coxiella burnetii* was detected in ticks collected from camels (*Hy. dromedarii* and *Hy. anatolicum*) in the district of Lakki Marwat, wild mice (*Ha. cornupunctata*) in Charsadda, and goats and sheep (*Ha. cornupunctata*) in Dir Lower. *Coxiella* endosymbionts were detected in ticks collected from goats (*Ha. cornupunctata*) in district Dir Lower sheep (*Ha. montgomeryi* and *R. haemaphysaloides*) in Buner and Mansehra, and domestic fowls (*A. persicus*) in Chitral. *Coxiella* spp. were detected in ticks collected from goats (*Ha. bispinosa*) in the district of Dir Upper, from goats (*Ha. cornupunctata*) in Dir Lower, from goats and sheep (*Ha. cornupunctata* and *Ha. montgomeryi*) in Bajaur, and from goats (*Ha. bispinosa*) in Abbottabad (Table 4).

### Phylogenetic analyses of the obtained sequences

**Figure 2:**
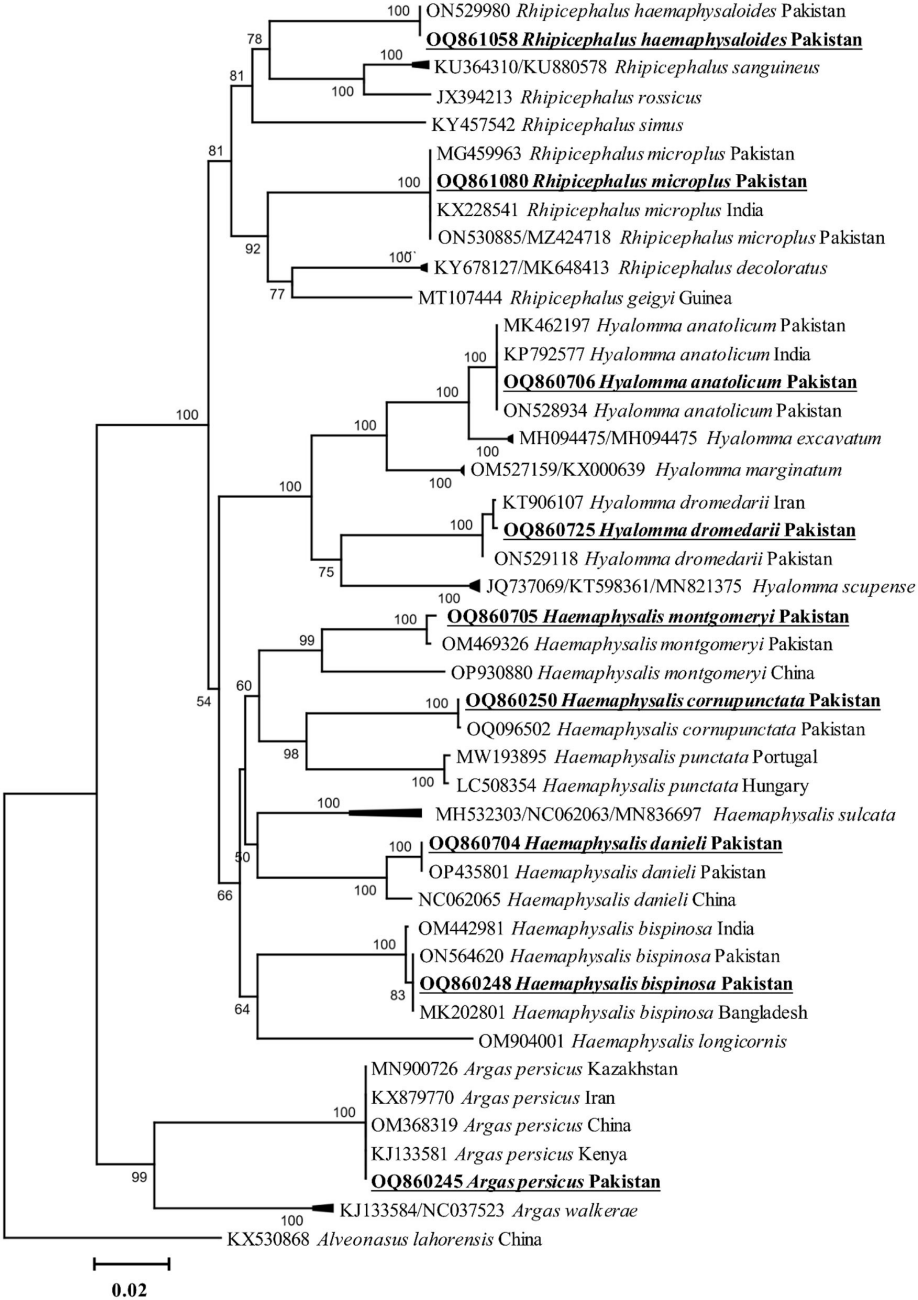
Phylogenetic tree based on *cox1* fragments of tick species. The sequence of *Alveonasus lahorensis* (KX530868) was used as an outgroup. The levels of bootstrap support (≥60%) for phylogenetic groupings are given at each node. The obtained sequences are represented with bold and underlined fonts.

All amplified PCR products were separately sequenced. The identical sequences were considered as a single consensus sequence. Trimmed and purified sequences of *cox1* fragments were obtained from nine tick species, including *Ha. cornupunctata, Ha. bispinosa, Ha. danieli, Ha. montgomeryi, Hy. anatolicum, Hy. dromedarii, R. haemaphysaloides, R. microplus*, and *A. persicus*. The BLAST results showed that the *cox1* fragments of the nine tick species were 99.6–100% identical to the corresponding species. Additionally, these sequences were phylogenetically clustered to the corresponding species reported from Pakistan, China, India, Bangladesh, Kazakhstan, Kenya, and Iran (Figure 2).

Based on the *GroEL* fragment, eight *GroEL* fragments of *Coxiella* spp. were detected in the aforementioned tick species except for *Ha. danieli* and *R. microplus*. By the BLAST results, the obtained *GroEL* fragment of *Coxiella* sp. (detected in *Ha. cornupunctata, Hy. dromedarii*, and *Hy. anatolicum*) showed maximum identity (99.8–100%) with the *C. burnetii* and phylogenetically clustered with the corresponding species reported from Slovakia (MG860513), China (ON455116), Russia (EF627450), USA (CP040059), and Thailand (MZ327921).

The obtained *GroEL* fragment of *Coxiella* spp. (detected in *Ha. cornupunctata, A. persicus*, and *R. haemaphysaloides*) showed 100% identity with the *Coxiella* endosymbiont reported from China (MZ367034 and MZ367036). Another *GroEL* fragment of *Coxiella* sp. (detected in *Ha. montgomeryi*) showed 99.8% maximum identity with the *Coxiella* endosymbiont reported from France (KP985488) and China (KP985490). Both partial fragments clustered with the corresponding *Coxiella* endosymbiont in the phylogenetic tree.

**Figure 3:**
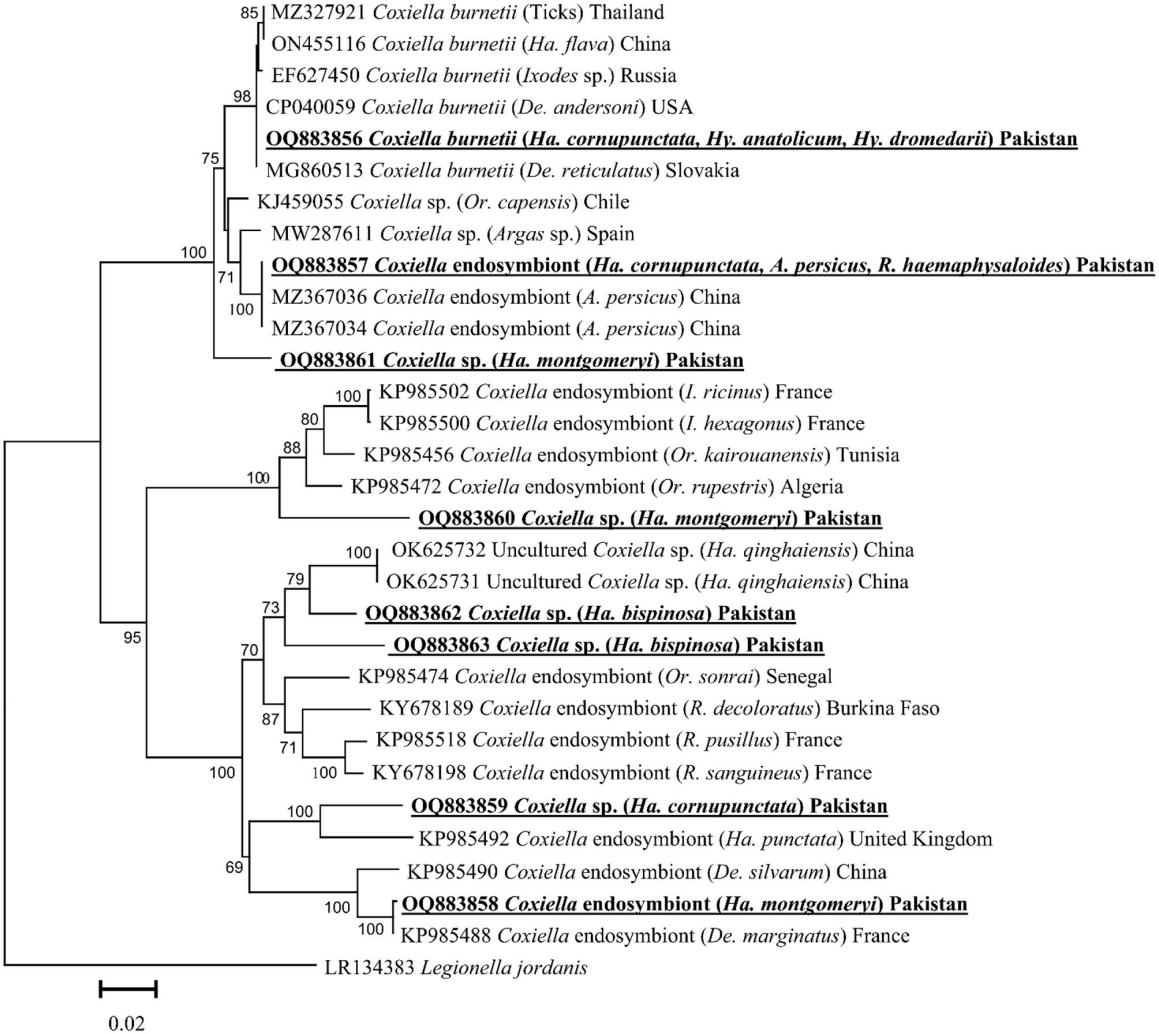
Phylogenetic tree based on *GroEL* fragments of *Coxiella* spp. detected in tick species. The sequence of *Legionella jordanis* (LR134383) was used as an outgroup. The levels of bootstrap support (≥ 60%) for phylogenetic groupings are given at each node. The obtained sequences are represented with bold and underlined fonts.

The BLAST results of the obtained *GroEL* fragments of *Coxiella* sp. OQ883859 (detected in *Ha. cornupunctata*) showed 88–90% maximum identity with *Coxiella* endosymbiont reported from the United Kingdom (KP985492), *Coxiella* sp. OQ883860 (detected in *Ha. montgomeryi*) showed 90.23% identity with *Coxiella* sp. reported from France (KP985502 and KP985500), Tunisia (KP985456), and Algeria (KP985472). The *Coxiella* sp. OQ883861 (detected in *Ha. montgomeryi*) showed 95.4% identity with *Coxiella* sp. reported from Chile (KJ459055) and Spain (MW287611), while *Coxiella* sp. OQ883862 (detected in *Ha. bispinosa*) showed 93.9% identity with *Coxiella* sp. reported from China (OK625731 and OK625732), and *Coxiella* sp. OQ883863 (detected in *Ha. bispinosa*) showed 91.3% identity with *Coxiella* sp. reported from China (OK625731 and OK625732). In the phylogenetic tree, all the obtained aforementioned *Coxiella* sp. sequences were clustered to the corresponding *Coxiella* sp. sequences (Figure 3).

The obtained *cox1* partial fragments of ticks were submitted to GenBank under accession numbers: OQ860250 (*Ha. cornupunctata*), OQ860248 (*Ha. bispinosa*), OQ860704 (*Ha. danieli*), OQ860705 (*Ha. montgomeryi*), OQ860706 (*Hy. anatolicum*), OQ860725 (*Hy. dromedarii*), OQ861058 (*R. haemaphysaloides*), OQ861080 (*R. microplus*), and OQ860245 (*A. persicus*). The obtained *GroEL* partial fragments of *Coxiella* spp. were submitted to GenBank under accession numbers: OQ883856 (*C. burnetii*), OQ883857 (*Coxiella* endosymbiont), OQ883858 (*Coxiella* endosymbiont), OQ883859 (*Coxiella* sp.), OQ883860 (*Coxiella* sp.), OQ883861 (*Coxiella* sp.), OQ883862 (*Coxiella* sp.).

## Discussion

Various ticks and their associated pathogens ideally propagate in Pakistan's humid and variable climatic conditions (Karim et al., 2017; Ali et al., 2019, 2020, 2021; Obaid et al., 2023). Microbiota belonging to different bacterial genera have been detected in different tick species in Pakistan (Karim et al., 2017; Ali et al., 2021; Alam et al., 2022; Khan Z. et al., 2022; Khan S. M. et al., 2023; Numan et al., 2022). Some serological surveys of Q-fever in small ruminants, large ruminants, rodents, and humans have been reported from Pakistan (Ahmed, 1987; Ali et al., 2022a). There is limited information available regarding the molecular characterization of *C. burnetii* and thus it remains an ignored zoonotic disease in the country. The association of *Coxiella* spp. with different ticks infesting various hosts has been reviewed globally (Guatteo et al., 2011); therefore, in this study, we summarized this association of *Coxiella* spp. with different ticks in Asia. Nine tick species including *Ha. cornupunctata, Ha. bispinosa, Ha. montgomeryi, Ha. danieli, Hy. anatolicum, Hy. dromedarii, R. haemaphysaloides, R. microplus*, and *A. persicus* infesting goats, sheep, camels, cattle, wild mice, and domestic fowls were genetically characterized. In addition, this is the first report regarding the molecular detection and phylogenetic positioning of *Coxiella* spp. associated with ticks in Pakistan. Overall, *C. burnetii*, two *Coxiella* endosymbionts, and five undetermined *Coxiella* sp. were genetically characterized based on *GroEL* fragments in various tick species.

Environmental factors such as humidity and temperature mainly affect the distribution of ticks, TBDs, and their zoonotic threats to human and animal health (Léger et al., 2013). Since the current study area's existing environmental and climatic conditions are favorable for tick infestation and propagation of various pathogens (Aiman et al., 2022; Ali et al., 2023), many ticks were collected during this survey. Contrary to previous studies, *Ha. cornupunctata* tick was more prevalent than other tick species such as *R. microplus* and *Hy. anatolicum* in Pakistan (Karim et al., 2017; Ali et al., 2019, 2021; Khan Z. et al., 2022). It may be due to examining different hosts, such as goats, sheep, and wild mice attributing a closed association with this species.

The age of the host is a significant factor to tick infestation. According to previous reports, a high tick burden was recorded on adult hosts compared with young ones (Ali et al., 2021; Kamran et al., 2021; Khan Z. et al., 2022). Large body surfaces and free grazing practices of adult animals make them more vulnerable due to high tick infestation. In contrast, the robust immune system, less grazing, and low body surface of the younger hosts contribute to less tick infestation (Swai et al., 2005). Female hosts were highly tick infested compared with the male hosts, which is consistent with previous findings (Ullah et al., 2023). Higher levels of progesterone and prolactin hormones in females make them susceptible to tick infestation (Anderson et al., 2013; Ahmed et al., 2023). The higher levels of progesterone and prolactin hormones may increase the susceptibility of females to tick's infections (Lloyd, 1983; Ahmed et al., 2023). Additionally, in the current study, ticks were predominantly reported in summer (June–August) compared with other seasons because the warm and humid climatic conditions in the region provide a suitable environment for the development of all stages of ticks (Ali et al., 2019, 2021). The comparatively wide host range noted for different *Haemaphysalis, Hyalomma*, and *Rhipicephalus* ticks may be due to frequent practices such as putting various hosts in the same shelter and over-crowded livestock and concurrent grazing in the survey area.

Major consequences have been revealed in the epidemiology of Q-fever upon the molecular detection of *C. burnetii* DNA in ticks collected from the environment, domestic and wild animals (Yessinou et al., 2022). It has been observed that ticks may transmit the Q-fever agent and pollute the environment as well as the host's body in Pakistan (Ullah et al., 2019). The association between different ticks and *C. burnetii* and its transstadially and transovarially transmission has been reported, suggesting the Q-fever transmission from infected to healthy animals through blood meal (Gong et al., 2020). In this study, molecular detection of *Coxiella* spp. varied in the aforementioned seven tick species collected from various hosts. A high prevalence of Q-fever in camels in this study may be attributable to the camels' vulnerability regarding *C. burnetii* infection or camel tick competence as a reservoir for this pathogen (Gumi et al., 2013). Common reservoirs for *C. burnetii* are small ruminants that may excrete a diverse number of these bacteria in their birth byproducts (placenta). *Coxiella* spp. were highly detected in ticks collected from small ruminants (goats and sheep), and these findings agreed with the previous serosurvey conducted in Pakistan (Ullah et al., 2019). *Coxiella* sp. detected in *A. persicus* ticks collected from domestic fowls suggest that different soft ticks may also be investigated as host reservoirs for various undetermined *Coxiella* spp., as reported in other studies (Trinachartvanit et al., 2018).

In the current study, phylogenetic analysis *via cox1* fragments of nine different tick species revealed a close evolutionary relationship with the same species reported from Pakistan, China, India, Bangladesh, and Iran, and these findings were supported by previous studies (Ahmad et al., 2022; Alam et al., 2022; Ali et al., 2022a; Khan S. M. et al., 2023). Phylogenetic analysis of *Coxiella* spp., detected in different tick species, showed close association with their respective species reported from the same or different tick species and humans. This association of *Coxiella* spp. may be due to the close interaction of infested animals with humans, which enhances zoonotic infections such as Q-fever in humans. So-far neglected surveillance of *Coxiella* spp. in the region demands immediate attention to its pathogenic consequences.

## Conclusion

*Coxiella* spp. were molecularly detected in ticks infesting goats, sheep, camels, cattle, wild mice, and domestic fowls and were confirmed through sequencing for the first time in Pakistan. Further research is essential to investigate any potential health risks due to these agents. The veterinarian livestock holders and farm workers lack knowledge regarding the epidemiology of Q-fever and its causative agents in Pakistan. Livestock holders should be adequately educated regarding Q-fever prevention and management practices because the occurrence of this agent can lead to long-term environmental contamination, which is a potential threat to animals and humans. Consequently, effective measures associated with Q-fever must be implemented, including limiting contact between herds, quarantining newly purchased animals, and using disinfectants that can reduce the spread of infection and possible transmission to humans.

### Institutional review board statement

The Advance Studies and Research Board (ASRB: Dir/A&R/AWKUM/2022/9396) of the Department of Zoology, Abdul Wali Khan University Mardan, Pakistan, approved prior consent for this study. Additionally, permission was taken from the owners of the animals to observe hosts and ticks collection. All the rules regarding animal welfare regulations were followed while handling the animals.

## Data availability statement

The datasets presented in the study have been deposited to the NCBI GenBank repository, accession numbers OQ860250 *(Ha. cornupunctata*), OQ860248 (*Ha. bispinosa*), OQ860704 (*Ha. danieli*), OQ860705 (*Ha. montgomeryi*), OQ860706 (*Hy. anatolicum*), OQ860725 (*Hy. dromedarii*), OQ861058 (*R. haemaphysaloides*), OQ861080 (*R. microplus*), OQ860245 (*A. persicus*), OQ883856 (*C. burnetii*), OQ883857 (*Coxiella* endosymbiont), OQ883858 (*Coxiella* endosymbiont), OQ883859 (*Coxiella* sp.), OQ883860 (*Coxiella* sp.), OQ883861 (*Coxiella* sp.), and OQ883862 (*Coxiella* sp.).

## Ethics statement

The animal study was reviewed and approved by the Advance Studies and Research Board (ASRB: Dir/A&R/AWKUM/2022/9396) of the Department of Zoology, Abdul Wali Khan University Mardan, Pakistan, approved prior consent for this study. Written informed consent was obtained from the owners for the participation of their animals in this study.

## Author contributions

AAli designed the study. AAli, MMA, TT, SBK, and AAlo carried out the experiments of the study. AAli, MN, ZUI, GR, MKO, SBK, and SU collected the tick samples and performed the experiments. AAli, MKO, and MN performed the phylogenetic and statistical analyses. All authors have read and agreed to the published version of the manuscript.
